# The Role of Alternative Splicing in the Control of Immune Homeostasis and Cellular Differentiation

**DOI:** 10.3390/ijms17010003

**Published:** 2015-12-22

**Authors:** Mehmet Yabas, Hannah Elliott, Gerard F. Hoyne

**Affiliations:** 1 Department of Immunology and Infectious Disease, the John Curtin School of Medical Research, the Australian National University, Acton 2601, Australia; mehmet.yabas@anu.edu.au; 2 Department of Genetics and Bioengineering, Faculty of Engineering, Trakya University, Edirne 22030, Turkey; 3 School of Health Sciences, University of Notre Dame Australia, Fremantle 6959, Australia; hannah.elliott1@my.nd.edu.au; 4 Institute of Health Research, University of Notre Dame Australia, Fremantle 6959, Australia; 5 School of Medicine and Pharmacology, University of Western Australia, Nedlands 6009, Australia

**Keywords:** apoptosis, B cells, T cells, hnRNP proteins, immune tolerance, pre-mRNA alternative splicing

## Abstract

Alternative splicing of pre-mRNA helps to enhance the genetic diversity within mammalian cells by increasing the number of protein isoforms that can be generated from one gene product. This provides a great deal of flexibility to the host cell to alter protein function, but when dysregulation in splicing occurs this can have important impact on health and disease. Alternative splicing is widely used in the mammalian immune system to control the development and function of antigen specific lymphocytes. In this review we will examine the splicing of pre-mRNAs yielding key proteins in the immune system that regulate apoptosis, lymphocyte differentiation, activation and homeostasis, and discuss how defects in splicing can contribute to diseases. We will describe how disruption to *trans*-acting factors, such as heterogeneous nuclear ribonucleoproteins (hnRNPs), can impact on cell survival and differentiation in the immune system.

## 1. Introduction

The immune system has evolved to respond to a myriad of pathogens. It is composed of two effector arms known as the innate and adaptive immune systems and they have specialized roles to play in protecting the host from infectious diseases and maintaining long-term health [1,2]. The innate immune system is composed of physical barriers such as the skin and mucous membranes but it is also composed of specialized cells such as macrophages, dendritic cells and granulocytes including neutrophils, basophils and mast cells. Many of these cells have a phagocytic function to help remove cellular debris and pathogens to maintain tissue homeostasis. These innate immune cells play a critical role to trigger adaptive immune responses [1,2]. Importantly these innate immune cells lack expression of antigen specific receptors but instead rely on expression of a range of evolutionarily conserved pattern recognition receptors to identify microbial derived components including proteins, lipids, carbohydrates and nucleic acids [3].

The adaptive immune system is composed of T and B lymphocytes and they are able to generate specific immune responses to defined antigens, and can generate immunological memory. B cell lineage differentiation occurs in the bone marrow and mature B cells are the major antibody producing cells of the immune system. B cells express an antigen-specific and clonally restricted receptor known as an immunoglobulin (Ig) molecule or B cell receptor (BCR). T cell lineage differentiation is restricted to the thymus and as they mature T cells express a clonally restricted T cell receptor (TCR) and develop into two major lineages referred to as TCRαβ^+^ and TCRγδ^+^. The TCRαβ^+^ lineage represents the major population of T cells in the peripheral immune system comprising >90% of the total T cells. These cells can give rise to two distinct subsets referred to as CD4^+^ T_helper_ (T_h_) cells which help B cells to produce antibodies and CD8^+^ cytotoxic T cells (CTLs) which help to recognize and destroy virus infected cells and tumor cells. In contrast, the TCRγδ^+^ cells play a role in immune surveillance in a range of tissues. In addition to TCRαβ^+^ and TCRγδ^+^ T cells, Natural Killer T (NKT) cells and NK cells also arise during thymic development.

Alternative splicing of pre-mRNA is prevalent in mammalian genomes and acts as a mechanism to amplify genetic diversity by enabling the number of proteins arising from the genes to be increased by a factor of 10 times [4]. Alternative pre-mRNA splicing can also regulate protein expression in a cell-specific or tissue-specific manner in response to precise environmental or developmental cues. Multiple variants of protein (isoforms) can be produced from a single gene [5,6]. Alternative splicing offers flexibility to the transcriptome and proteome to help fine tune important and complex cellular responses such as regulation of cell viability, differentiation and apoptosis in response to environmental cues [7]. Mutations that disrupt the process of alternative splicing from pre-mRNAs to proteins can be deleterious to health and readers are referred to a more comprehensive review on the topic by Cooper, Wan and Dreyfuss [8].

The immune system must be able to distinguish between self and non-self to protect against the risk of autoimmunity, and this helps to mount an appropriate immune response to neutralize and remove infectious (non-self) agents that cause disease. The immune system has to balance the need to remove autoreactive T and B cells during their development that express self-reactive receptors and to maintain a sufficiently diverse lymphocyte repertoire to be able to respond to any infectious agent. One of the best examples of alternative splicing in the immune system is the generation of the diverse repertoire of antigen receptors expressed by B and T cells that can recognize a wide range of protein antigens [9,10]. Apoptosis is a critical mechanism for regulation of immune tolerance to self-antigens. The immune system also uses apoptosis to regulate the turnover of lymphocytes throughout life which is referred to as lymphocyte homeostasis, and is vital in the resolution of immune responses to kill off effector cells once a pathogen has been cleared [11]. It has emerged in recent years that a number of the proteins involved in regulation of apoptosis from cell surface receptors to signaling proteins involved in the effector pathway of cell death are controlled at the level of alternative splicing of pre-mRNA. Therefore, in this review we will examine the key role for apoptosis regulators and highlight where defects in alternative splicing can have important consequences on the decision between immunity and tolerance and on lymphocyte homeostasis. We will also examine the role of specific heterogeneous nuclear ribonucleoproteins (hnRNPs) in the regulation of pre-mRNA alternative splicing to illustrate how these proteins can impact on immune cell development and function.

## 2. Alternative Splicing of Pre-mRNA

**Figure 1:**
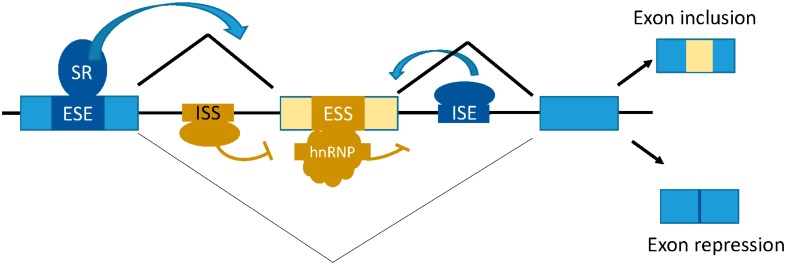
Mechanism of alternative splicing of pre-mRNAs. The *cis-*regulatory elements that control alternative splicing are composed of unique nucleotide sequences. The exonic splicing enhancers (ESE) and intronic splicing enhancers (ISE) (dark blue) respectively promote exon inclusion (light blue boxes), while exon repression requires the exonic splicing silencer (ESS) and intronic splicing silencer (ISS) elements (brown). The *trans*-acting factors that promote exon inclusion are the Serine (S)-Arginine (R) SR rich proteins while hnRNP proteins can promote exon skipping. In the diagram the middle exon can be included to give rise to a polypeptide with 3 exon units, while the alternatively spiced polypeptide skips the variable exon to yield a protein with 2 exon units.

Alternative pre-mRNA splicing considerably expands the genome’s “vocabulary” of variant genes, resulting in different functions and outcomes within the cell [6]. As a result metazoans depend on alternative splicing as their major contributor to protein diversity [5,7]. Alternative splicing results in the rearrangement of exons within a multi-exon pre-mRNA. Specific internal “cassette” exons can be spliced out or included in the final mRNA [5] (Figure 1). The mRNA transcript can also be rearranged by altering exon splice sites (SS) resulting in a lengthened or shortened exon [5]. Both the 5′ and 3′ SS can be altered. There can be alternative usage of 5′ or 3′ untranslated regions (UTRs) that can influence whether or not regulatory sequences are present for a splicing factor to bind or they may contain sequences which promote binding of microRNAs [12,13]. Collectively these modifications can influence the stability expression and translation of specific mRNA transcripts. 5′ UTRs are altered with the use of alternative promoters and splicing and 3′-terminal exons are altered by splicing an alternative polyadenylation site [5]. The failure to remove introns, known as intron retention, is another mechanism that results in alternative splicing [5].

The process of intron removal and exon joining is catalyzed by the enzymatic complex referred to as the spliceosome. This is a multiprotein complex that is assembled *de novo* on pre-mRNA transcripts. Nuclear pre-mRNA splicing involves two simple ATP-dependent chemical reactions known as SN2-type *trans*-esterification reactions, which involve three reactive regions in the pre-mRNA [14]. First the branch point sequence (BPS) in the intron breaks the phosphodiester bond at the 5′ SS. This generates a free 5′ exon and a structure known as an intron lariat-3′ exon. A second phosphodiester bond is broken at the 3′ SS by the 3′ hydroxyl of the 5′ exon resulting in exon ligation and excision of the lariat intron. The folding of nuclear pre-mRNA introns to enable splicing is dependent on *trans*-acting factors that comprise the spliceosome. This dependency does not occur in self-splicing group II introns that are able to adopt a three-dimensional fold for splicing by themselves [15]. The human spliceosome consists of a single non coding RNA (snRNA) that contains uridine-rich small nuclear ribonucleoproteins (snRNPs) named U1, U2, U4/U6 and U5 and ~45 distinct snRNP-associated proteins [16]. In addition to snRNP associated proteins, studies have shown that there are approximately 170 spliceosome-associated factors with individual assembly intermediates each containing around 125 proteins (see review by Wahl, Will and Luhrmann for the different spliceosome assemblies and conformations that occur during splicing [16]).

Many factors determine whether or not a pre-mRNA sequence will be alternatively spliced. For example, the inclusion or exclusion of an exon is determined by the ability of the spliceosome to recognize the exon’s surrounding SS that are determined by *cis* elements that help in regulating SS selection by modulating the interaction with snRNP and its substrate. The recognition of the spliceosome to the exon’s SS results in the formation of the exon-definition complex [16]. Different factors can inhibit or enhance spliceosomal recognition of these SS, with splicing regulatory sequences more commonly found in introns rather than exons [5,17]. Binding sites for regulators are often found immediately adjacent to the BPS or 5′ SS or within the polypyrimidine tract [5]. Some regulatory sequences are capable of regulating exons hundreds of nucleotides away from their location, such as the dsx repeat element (dsxRE), a splicing enhancer found in *Drosophila melanogaster* [18]. *Cis*-regulatory sequences referred to as intronic and exonic splicing enhancers (ISE and ESE) can promote or intronic and exonic splicing silencers (ISS and ESS) can suppress the assembly of the snRNP on the substrate (Figure 1). There are two protein families of *trans*-acting regulatory proteins the Serine (S)-Arginine (R) rich (SR) and the hnRNPs that act to recruit the spliceosome machinery to the SS [19,20,21] (Figure 1). The SR proteins tend to enhance SS usage whereas hnRNP proteins favor exon skipping (Figure 1), but this is not a strict rule as there are examples which are contrary to this generalization [22,23,24]. Both families of RNA binding proteins can exhibit enhancing and silencing activity when bound to an intron [25]. The ISE and ESE elements are found in both constitutive and cassette exons and appear to be a means of defining exons [21,26].

The relative affinity of the 5′ SS and 3′ SS determines the particular snRNP that will bind to the specific SS. This plays an important role as the consensus sequences that make up the 5′ SS, 3′ SS and BPS are degenerate in higher eukaryotes where alternative splicing is predominant [5]. This also results in SS alone not being capable of efficiently directing spliceosomal assembly. SR and hnRNP proteins are therefore required to manage and direct the spliceosome [5]. SR proteins bind with low affinity and specificity to both the pre-mRNA and other Arginine (R)-Serine (S) (RS) domain containing spliceosomal proteins. Interestingly, SR proteins for the most part are absent in yeast with exceptions such as the SR-like protein Npl3 [27]. The addition of SR proteins in metazoans correlates with the degeneration of the consensus sequences defining the 5′ SS, 3′ SS and BPS [5]. They also share a common domain structure consisting of one or two RNA recognition motifs (RRMs) followed by a RS domain containing RS dipeptides [19]. These serine dipeptides can be highly phosphorylated [28]. High throughput sequencing of chromatin associated RNA in *Drosophila* revealed that splicing of genes can occur in a co-transcriptional manner and the efficiency of the co-transcriptional splicing varies between genes [29]. The variability in efficiency of co-transcriptional splicing is thought to be regulated at the level of the intron and is mediated by different RNA binding proteins such as SR and hnRNP proteins [29]. Furthermore, there is a direct correlation between those genes where there is no co-transcriptionally splicing with annotated alternative exons. [29,30,31].

Alterations to the activity of splicing factors can be influenced by posttranslational modification [28,32]. In a large scale phosphoproteomic analysis of TCR signaling in the Jurkat T cell line Mayya *et al.* [33], identified that splicing factors and components of the spliceosome can be targets of TCR induced phosphorylation. This is important as both hypo- and hyper-phosphorylation of SR proteins *in vitro* has been demonstrated to modulate protein function [28,34]. These posttranslational modifications can influence protein-protein interactions as well as protein–RNA interactions to regulate their activity and to direct expression of different isoforms of proteins with different functions that derive from a common pre-mRNA. The hnRNP proteins hnRNPLL and CELF2 function in alternative splicing and expression of both proteins is linked to TCR signaling [35,36]. In the following discussion we want to explore the role of a range of well characterized RNA binding proteins that are involved in alternative splicing in the immune system.

## 3. HnRNP Proteins and Their Role in Pre-mRNA Alternative Splicing

Alternative splicing plays an important role to alter the biological function of key immune proteins and this can impact on the host’s immune response [37]. One of the first genes identified in B and T cells to undergo alternative splicing was the *Ptprc* encoding the CD45 protein which is an abundant tyrosine phosphatase found on the surface of most leukocytes [38,39,40,41]. CD45 regulates TCR signaling by dephosphorylating two regulatory tyrosine residues (Lck Y505 and Lck Y394) on the Src kinase p56^lck^ [20,42,43]. In addition to its tyrosine phosphatase function it has also been shown to have an important role in cell survival and death [38,44,45]. CD45 is expressed specifically on all nucleated hematopoietic cells and their precursors and comprises up to 10% of cell surface protein in T and B cells [46]. The *Ptprc* gene has three variable cassette exons (exons 4, 5 and 6), which encode the CD45RA, RB and RC segments that undergo extensive posttranslational modification by the addition of numerous *O*-glycosylation sites within the extracellular domain [47]. These different CD45 isoforms are used to distinguish recently activated and antigen-experienced cells from naïve T cells [40,45]. In B lymphocytes *Ptprc* is spliced to include all three exons and is termed B220 or CD45RABC [48]. Naïve T cells express CD45 isoforms which include one (CD45RB) to two (CD45RAB/CD45RBC) of the cassette exons [40,45]. In contrast, memory T cells express the CD45RO isoform which lacks all three exons [40,45].

These different isoforms of CD45 are highly conserved and tightly regulated throughout development and are linked to activation status of the T cell [38,40,49,50]. There are functional differences between the different isoforms with the lower molecular weight isoforms, such as CD45RO, being more prone to homodimerization and subsequent auto-inhibition resulting in T cell signaling attenuation than the higher molecular weight isoforms [50,51]. Absence of CD45 isoforms on the cell surface results in impairment of both T and B cell activation and subsequent proliferation and cytokine production [46,48,52]. As a result their expression is tightly regulated during development and is linked with cellular activation in response to antigen receptor signaling [53]. A range of different hnRNP proteins, which will be discussed further below, have been shown to play a role in alternative splicing of CD45 pre-mRNA and are dependent on TCR dependent activation of Protein Kinase C PKC and Ras [54]. The hnRNP proteins act as ESS-responsive negative regulators of splicing and are thus antagonistic to SR proteins [55,56,57]. Changes in the level of endogenous hnRNP proteins can mediate important pre-mRNA splicing modifications that result in different cell functions [55].

### 3.1. HnRNPL

**Figure 2:**
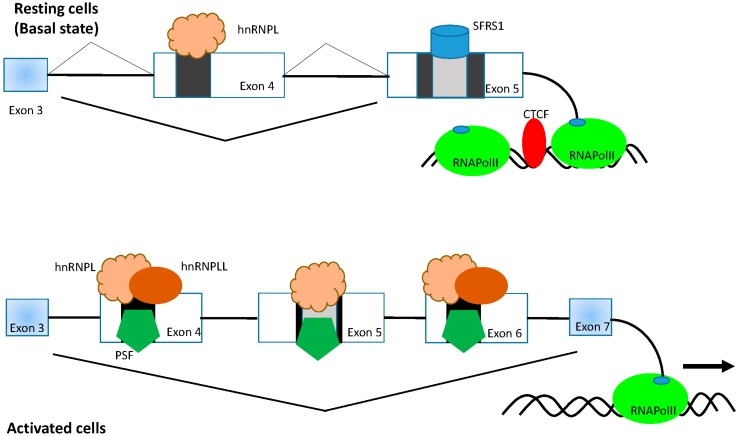
Alternative splicing of the *Ptprc* gene. The *Ptprc* gene can give rise to multiple isoforms through alternative splicing of pre-mRNA that involves three variable exons 4, 5 and 6. All three variable exons contain the ARS motif (black) and the ESE (black). The upper panel indicates the conditions of alternative splicing in T cells in the basal resting state. The hnRNPL protein mediates basal repression of exon 4 in resting cells, while the SR protein SRSF1 mediates the inclusion of exon 5. In addition, the CTCF protein can bind to the ESE sequence of exon 5 to mediate pausing of the RNAPII elongation to promote exon 5 inclusion. The bottom panel shows the alternative splicing of *Ptprc* gene following TCR activation. The proteins PSF and hnRNPLL are recruited to the exon 4 and exon 6 ARS together with hnRNPL to promote repression of both exons, while hnRNPL and PSF mediate repression of exon 5.

HnRNPL is an RNA binding protein that has four RRM domains and can act as both a splicing enhancer as well as splicing silencer. In addition to its role in regulating inclusion of cassette exons it can also function in intron retention and polyadenylation site choice [24,58,59,60]. The hnRNPL protein was one of the first *trans*-acting factors identified in regulating the splicing of the three variable exons (4, 5 and 6) of the *Ptprc* gene. Splicing of the three variable exons of CD45 was shown to be linked to TCR signaling in naïve T cells and is dependent on the presence of an activation response sequence (ARS) within each of the exons of the variable exons [50,60] (Figure 2). The ARS motif is located within a 60 nucleotide sequence of the ESS1 of exons 4 and 6. Identification of the ARS motif helped to identify proteins that are involved in regulation of splicing of these three variable exons. HnRNPL was identified as the basal mediator of repression of *Ptprc* variable exons [24,50,60,61] (Figure 2). Thymocytes from *Hnrnpl*^−/−^ mice show increased inclusion of the CD45 exons that helped to confirm the important role for the hnRNPL protein *in vivo* for CD45 exon repression [62]. The *Hnrnpl*^−/−^ mice were healthy but revealed an arrest in thymocyte differentiation at the double negative to double positive transition that in turn leads to a marked decrease in thymic cellularity.

### 3.2. HnRNPLL

The hnRNPLL protein has four RRM domains and is upregulated following TCR signaling in resting T cells and is also highly expressed in memory T cells [31]. The functional role for hnRNPLL in T cells has been uncovered through the use of cell based assay systems as well as studies with a novel mouse strain that expresses a loss-of-function allele of *Hnrnpll* generated by *N*-ethyl-*N* nitrosourea (ENU) mutagenesis in mice [26]. Topp *et al.* [61] identified hnRNPLL as a protein that bound to the ARS motif of exon 4 to mediate its repression while in an alternative approach Oberdoerffer *et al.* [63] used an shRNA genetic screen to identify hnRNPLL as an important factor that regulates repression of exon 4 of CD45 (Figure 2). The studies by Wu *et al.* [26] identified a novel ENU induced allele in a mouse strain caused by a point mutation V136D within the RRM1 domain and this led to destabilization of the domain leading to a loss of protein function. In contrast to hnRNPL, the hnRNPLL protein does not bind to the ARS motif in exon 5 of *Ptprc* and does not have any effect on the activation induced repression of exon 5 [24,63]. *Hnrnpll* expression is strongly induced following TCR activation and this leads to recruitment of the hnRNPLL protein to the ARS sequence of exon 4 and exon 6 where it can associate with hnRNPL leading to repression of the variable exons (Figure 2). Cho *et al.* [64], identified that hnRNPLL can induce a distinct pattern of intron retention surrounding target cassette exons in T cells. In particular three genes *Senp2*, *Ctse* and *Slc12a7* displayed greater intron retention in wild type cells compared to cells containing the hnRNPLL^thunder^ allele and in the wild type cells this correlated with exon inclusion. Furthermore, they validated the splicing of 15 genes by PCR and revealed 8 out of the 15 selected genes that displayed differential bands between wild type and hnRNPLL^thunder^ T cells (*Degs1*, *Sidt1*, *Mapkap3*, *Herc3*, *Ikbe*, *Cep110*, *Mllt6* and *Rap1gds1*) [64]. They believe that hnRNPLL can act as a splicing silencer and a splicing enhancer and this varies between different genes.

### 3.3. Polypyrimidine-Tract-Binding Protein (PTB)-Associated Splicing Factor (PSF)

The PSF protein is an hnRNP protein that contains two RRM domains and was originally identified as an essential *trans*-acting factor to regulate splicing of the variable exons of *Ptprc* [65,66]. PSF, like hnRNPLL, displays preferential binding to the ESS1 element of exon 4 of the *Ptprc* gene, but its association with the ESS1 motif is independent of both hnRNPL and hnRNPLL. PSF does not bind to the canonical ARS motif but to sequences outside of the core motif [61,66] (Figure 2). Recruitment of PSF to the CD45 ARS motif requires posttranslational modification of the protein via the serine/threonine kinase glycogen synthase kinase 3 (GSK3). In resting cells GSK3 phosphorylates PSF which facilitates its interaction with TRAP150, and the PSF-TRAP150 complex prevents PSF form binding to the ARS motif. Following T cell activation, the activity of GSK3 is inhibited which prevents phosphorylation of PSF and this allows recruitment of PSF to the ARS motif to mediate repression of the variable exons. Martinez and Lynch have proposed that the PSF-TRAP150 complex is likely to control a broader set of alternatively spliced pre-mRNAs in T cells [25].

### 3.4. Serine/Arginine-Rich Splicing Factor 1 (SRSF1)

The SRSF1 protein is a member of the SR protein family and it contains an N-terminal RRM-domain and a C-terminal RS rich domain which is thought to be involved in protein-protein interactions. The SRSF1 protein has been implicated in splicing of a number of immune pre-mRNAs including CD45 exon 5 and for the retention of an intron within the 3′ UTR region of the CD3ζ protein which is involved in the formation of the CD3 complex that is required for TCR signaling. SRSF1 can bind to the ESE sequence in exon 5 of the *Ptprc* gene. Exon 5 contains two ARS motifs which are separated by the ESE. SRSF1 can bind to the ESE and promote inclusion of exon 5 whereas hnRNPL can bind to the ARS sequence and mediate exon repression (Figure 2). It is believed that there is a balance in the levels of both SRSF1 and hnRNPL at the exon 5 region and depending on which protein is more abundant this will lead to inclusion or repression of exon 5.

In the context of CD3ζ the level of SRSF1 will determine whether there is intron inclusion or repression of a specific intron within the 3′ UTR of the *CD3ζ* (*Cd247*) gene [67]. The inclusion of the 3′ UTR intron leads to increased expression of the CD3ζ protein allowing for proper TCR signaling. When the intron within the 3′ UTR is repressed it leads to decreased CD3ζ protein expression and this can impact on TCR signaling and activation [67]. This could potentially have dangerous effects on immune tolerance as diminished TCR signaling could lead to escape of self-reactive lymphocytes during development. It has been noted that low levels of CD3ζ protein correlates with low levels of SRSF1 and development of the systemic autoimmune disease Systemic Lupus Erythematosus (SLE) [67].

### 3.5. The Src-Associated Protein in Mitosis of 68 kDa (Sam68)

Sam68 belongs to the signal transduction and activation of RNA metabolism (STAR) family of RNA binding proteins that plays a crucial role in regulating cell proliferation, survival and differentiation across species [68]. The STAR proteins have a KH (hnRNPK homology) domain embedded within a conserved region referred to as GSG (GRP33/Sam68/GLD1) which is required for protein–protein interaction and RNA binding. Sam68 has been shown to regulate alternative splicing of a couple of key target genes in T cells including *Cd44* and the generation of the anti-apoptotic isoform of the apoptotic regulator Bcl-X [69,70]. CD44 is widely expressed on lymphocytes where it functions in cell homing to lymphoid tissues but also plays a role through embryonic development and tumor progression [71,72,73,74]. There are ten variable exons within the *Cd44* gene of which exon 5 is the most intensively studied variable exon whose splicing is linked to TCR signaling pathways [73,75]. Ras-ERK signaling promotes interaction of Sam68 with the SR factor SRm160 to form a complex that is recruited to the ESE of exon 5 to promote exon inclusion [70]. This represents a prime example of how cell membrane signaling events in response to environmental cues, can modify protein expression within target cells through alternative splicing to facilitate changes in cell migration. The phosphorylation of Sam68 by ERK does not affect its affinity for RNA, but rather may be important in directing protein-protein interactions that facilitate SS recognition and/or spliceosome assembly [70].

In addition to its role in exon inclusion for CD44 exon 5, Sam68 can also bind to the SWI/SNF complex Brahma (Brm) protein to regulate the rate of RNA Polymerase II (RNAPII) elongation of mRNA transcripts. Batsche *et al.* [76] identified that Sam68 and the Brm protein can associate on CD44 exon 5 in response to phorbol 12-myristate 13-acetate stimulation in HeLa cells. The association of Sam68 with Brm is thought to induce pausing of RNAPII in the variant region of *Cd44* exons 4–6. This mechanism could facilitate inclusion of alternative exons within weak SS’s as the slowing of RNAPII elongation rates can influence alternative splicing across species [77,78].

Sam68 has been shown to be involved in alternative splicing of the *Bcl2l1* gene which encodes the Bcl-X protein [79]. The *Bcl2l1* gene has two 5′ SS that are located at a proximal and distal region within exon 2 of the gene. Depending on which variable SS is used it can yield alternatively spliced transcripts of the Bcl-X protein that have distinct effects on cell survival [11]. The long form of Bcl-X (Bcl-X_L_) has an anti-apoptotic role in lymphocytes and is induced in response to TCR and growth factor signaling pathways by splicing from the proximal 5′ SS of the *Bcl2l1* gene. Alternatively if cells select the distal 5′ SS this leads to generation of the small isoform Bcl-Xs and which has potent apoptotic activity resulting in cell death [69,80]. Sam68 activity favors the selection of the distal region of exon 2 leading to formation of the Bcl-Xs isoform that induces apoptosis [81]. More recently Bielli *et al.* [82], identified a novel transcript factor FBI-1 through a yeast two hybrid screen for proteins that interact with Sam68. FBI-1 can interact directly with Sam68 and affects its interaction with *Bcl2l1* pre-mRNA to promote splicing of the anti-apoptotic Bcl-X_L_ isoform to protect cell from apoptosis.

### 3.6. CCCTC Binding Factor (CTCF)

CTCF is a Zinc finger DNA binding protein that was found to bind to exon 5 of *Ptprc* in cells that abundantly express the phosphatase enzyme. As mentioned above one of mechanisms that can promote inclusion of weak upstream exons is the stalling of RNAPII to favor exon inclusion [77,83]. CTCF binding to exon 5 promoted RNAPII pausing upstream of CTCF binding sites and its binding to the exon 5 site can be inhibited by DNA methylation [84]. This was one of the first documented studies to show how a protein, normally associated with DNA methylation, could be recruited into the process of alternative splicing.

### 3.7. T cell Restricted Intracellular Antigen 1 (TIA-1)

TIA-1 is an RNA binding protein that contains three RRM domains and a glutamine-rich C-terminal domain. TIA-1 and the related protein TIAR have been implicated in cellular and virus induced apoptosis [85,86], and also play a role in RNA processing to help silence tumor necrosis factor (TNF)-α expression [87]. Both proteins also appear to be involved in regulating the skipping of exon 6 on the *Fas* gene [88]. The *Fas* exon 6 encodes the transmembrane domain of the protein and thereby skipping of this exon gives rise to a soluble version of the FAS receptor which plays an important physiological role to inhibit FAS signaling [89,90]. TIA-1 binds to a uridine rich sequence in the exon 6 5′ SS to promote exon 6 inclusion [88]. TIA-1 activity is regulated by posttranslational modification by the FAS activated serine threonine kinase (FASTK) which is activated in response to FAS ligation in T cells. Increased FASTK activity promotes TIA1 to induce exon 6 inclusion of FAS leading to increased production of the full length transmembrane protein FAS that would facilitate cell death. Izquierdo *et al.* [91], demonstrated that FASTK activity on *Fas* alternative splicing required functional TIA-1 and TIAR proteins as splicing was diminished in the absence of both proteins and was disrupted by a mutation in the *Fas* exon 6 5′ SS ISE.

## 4. Immunity *versus* Tolerance

The immune system has evolved to recognize and destroy foreign microbes and the hallmark of the adaptive immune system is its ability to recognize and respond to specific antigens and to generate long lived memory responses [92]. T and B cells are the major effector cells of the adaptive immune response and the immune system has a diverse repertoire of both cell types to enable the host to fight infections caused by pathogenic microorganisms. As mentioned T and B lymphocytes express a clonally restricted antigen specific receptor and during lymphocyte differentiation these receptors are tested for their reactivity to self-antigens. If the antigen receptor displayed on the surface of lymphocytes has high affinity for self-antigens the cell will be instructed to undergo apoptosis [92]. This favors the maturation of T and B cells that express antigen receptors with low to moderate affinity for specific antigen. The selection of antigen specific T and B cells is referred to as central tolerance and occurs during the process of both T cell development in the thymus and in the bone marrow for B cells. The development of T and B cells is critical to prevent autoimmunity and autoimmune diseases. In the following section we would like to highlight a few salient examples where alternations in alternative splicing can have an important impact on the outcome of fundamental processes within the immune system that control apoptosis, lymphocyte homeostasis and B cell responses.

## 5. Control of Apoptotic Effector Proteins by Alternative Splicing

The strength of TCR or BCR signaling in developing lymphocytes is crucial in determining the outcome of central tolerance as is the ability to eliminate autoreactive lymphocytes by apoptosis [93,94]. Much has been learnt about the proximal and distal biochemical signaling pathways that emanate from the TCR and BCR. These studies have identified a number of cell surface proteins that facilitate the initial stages of antigen receptor signaling and are crucial for transducing signals from the membrane into the cytoplasm and nucleus to regulate gene expression [95]. A number of key factors have been identified that control the choice between immunity and tolerance at the level of cell survival. Some of these factors require pre-mRNA alternative splicing that give rise to specific protein isoforms. If the splicing of a key target gene is disrupted in any way it can have an important impact on the final outcome of health and disease.

A number of apoptotic regulators have been shown to undergo alternative splicing [32]. Proteins that belong to the TNF superfamily function as death receptors or death ligands to induce apoptotic signaling pathways to mediate cell death [11,96]. These proteins play important roles in variety of setting including embryonic development, in the immune system to regulate immune homeostasis and the resolution of immune responses following pathogen clearance. One of the death receptors widely expressed by lymphocytes include the FAS receptor (CD95) which interacts with the FAS ligand (FASL, CD95L). A soluble form of FAS can be generated by alternative splicing via exon skipping of the variable exon 6 of the *Fas* gene that lacks the transmembrane domain to yield a soluble FAS protein which can inhibit apoptosis via the FAS receptor pathway [97,98]. As mentioned above the TIA-1 and TIAR hnRNP proteins are key *trans*-acting factors that regulate exon 6 inclusion to mediate expression of the full length FAS protein [88]. The activity of TIA-1 is controlled through a feed-forward regulatory pathway where by activation of FAS signaling in the T cell leads to increased activity of FASTK which phosphorylates TIA-1. This facilitates the recruitment to the exon 6 ISE to mediate exon inclusion [91]. A number of studies have identified different spliced isoforms of the FAS in activated human peripheral blood mononuclear cells and in T cell tumor lines [97,98]. Further highlighting the important role for the regulation of pre-mRNA splicing of death receptor proteins is the observation that elevated levels of soluble FAS are detected in the serum of patients with the autoimmune disease SLE and other malignancies [90].

The induction of apoptosis is dependent on a well-conserved signaling pathway that leads to inhibition of pro-survival proteins such as Bcl-2 and Bcl-X_L_, but upregulation of a range of pro-apoptotic proteins such as the Bcl-2-like 11 protein (also known as Bcl-2-interacting mediator of cell death, Bim), Bcl-2 associated X protein (Bax) and Bak that lead to apoptosis and cell death [11]. As discussed above the Bcl-X_L_ protein functions to promote cell survival like Bcl-2, but the *Bcl2l1* gene can be alternatively spliced to yield different isoforms with distinct biological activities. The Sam68 protein promotes the use of the distal 5′ SS to yield the Bcl-Xs isoform [69]. In addition, exposure of cells to ceramide, a known proapoptotic inducer, can preferentially promote splicing of the Bcl-Xs isoform to promote cell death [69,99,100,101]. In contrast, the FBI protein can interact with Sam68 to promote splicing of the *Bcl2l1* gene from the proximal 5′ SS to give rise to the long isoform Bcl-X_L_ that protect cells from apoptosis.

The *Bcl2l11* gene, encoding another pro-apoptotic member Bim, undergoes pre-mRNA alternative splicing to yield three different isoforms (Bim_S_, Bim_L_, Bim_EL_) that differ in their capacity to induce apoptosis [102,103,104]. Bim plays a critical role in inducing cell death in the thymus and is required to eliminate self-reactive T cells [102]. The most effective inducer of apoptosis is the Bim_S_ isoform, while Bim_L_ and Bim_EL_ have the least apoptotic activity [104,105]. Analysis of *Bim^−/−^* mice revealed that these animals contained 2–3 times more T and B cells compared to wild type littermates and displayed lymphadenopathy with increasing age. *Bim^−/−^* mice also developed higher levels of autoreactive antibodies with an increase in autoreactive IgG antibodies to nuclear antigens and deposits of immune complexes in the kidneys consistent with a Lupus like autoimmune disease [105]. These data highlight that the levels of Bim are important for maintaining lymphocyte homeostasis and to protect against autoimmunity.

Both *cis*-elements and *trans*-acting factors contribute to pre-mRNA alternative splicing of *Bcl2l11* that promotes inclusion of either *Bcl2l11* exons 3 or 4 and this can give rise to two distinct mRNA isoforms. Exons 3 and 4 cannot be spliced together because exon 3 contains a functional polyadenylation signal and lacks a functional 5′ SS. Exon 3 containing splice variants are not proapoptotic because they lack the BH3 domain encoded in exon 4 which is involved in interacting and antagonizing the pro-survival members of Bcl-2 family [104,106]. Overexpression of the splicing factor SRSF1 promotes inclusion of exon 3 over exon 4 and this favors the expression of non-apoptotic splice variants of Bim in mammary epithelial cells [106]. In addition, a single nucleotide polymorphism (SNP) in exon 4 of *Bcl2l11* can cause exon skipping of exon 3 that is dependent on *trans*-acting factors PTBP1 and hnRNPC [107] and the splicing factor SRSF6 [108].

## 6. Alternative Splicing and Control of T Cell Activation

Control of T cell activation is crucial to prevent inappropriate activation of mature effector T cells. The CD28 receptor expressed on TCRαβ^+^ cells is involved in costimulation of T cell responses to drive full activation of the cell. The CTLA-4 protein is upregulated on activated T cells and has been shown to play a critical role in dampening TCR signaling in activated T cells and inhibit cellular proliferation and function [109,110,111,112]. The CTLA-4 receptor has 4–5-fold higher affinity for the CD80/CD86 ligands expressed by professional antigen presenting cells than that of the costimulatory receptor CD28 [111,112].

Recent studies by Butte *et al.* [113], identified that T cell activation via TCR and CD28 signaling leads to increased splicing of a wide range of target genes. They found a range of target genes, some of which were known to be controlled by hnRNPLL [63] that underwent alternative splicing in response to TCR/CD28 signaling compared to TCR signaling alone [113]. This was the first report to demonstrate that costimulatory signals in T cells induce a program of alternative splicing. The costimulatory signal emanating from CD28 is crucial to prevent T cell clonal anergy in naive T cells following recognition of their antigen. Expression of the *Hnrnpll* gene was increased upon CD28 costimulation [113].

*Ctla4^−/−^* mice rapidly develop lymphoproliferative diseases with multi-organ infiltration and tissue destruction [114,115,116]. It is not surprising that the *Ctla4* gene has been identified as an autoimmunity susceptibility gene in a number of autoimmune diseases including type 1 diabetes, multiple sclerosis and Graves’ disease [117,118,119]. Mapping studies in humans identified a variation in the noncoding 3′ region of *CTLA4* which correlated with lower mRNA levels of the soluble form of CTLA-4 protein [119]. The novel splice variant termed liCTLA-4 lacked the exon 2 which encoded the CD80/CD86 ligand binding domain. It is believed that liCTLA-4 would act by inhibiting T cell activation and expansion and is therefore important for maintenance of immune tolerance and homeostasis and an equivalent spliced isoform was verified in mice [119,120]. However the specific *cis*-elements and *trans*-acting factors responsible for generating the spliced variant of CTLA-4 have yet to be described.

## 7. Control of T Cell Homeostasis

The immune system maintains tight control over lymphocyte numbers in the peripheral circulation that is determined by growth factor signaling through the common gamma chain (γ_c_) family of receptors predominantly IL-7, IL-15 and IL-2 [121]. Disruption to growth factor signaling through growth factor withdrawal can promote cell death by apoptosis. A failure to maintain stable numbers of lymphocytes in the immune system, especially CD4^+^ Foxp3^+^ regulatory T cells above a critical level can lead to a breakdown in self-tolerance and development of spontaneous autoimmunity [121]. The IL-7 receptor (IL-7R) is composed of IL-7Rα which helps to form the high affinity receptor, that pairs with the IL-2Rβ subunits and the γ_c_ chain. Signaling via the IL-7R is critical to maintain stable numbers of CD4^+^ and CD8^+^ T cells in the peripheral circulation of the immune system. The *Il7r* gene can undergo alternative splicing. For example the variable exon 6 that encodes the transmembrane domain of the receptor can be skipped to give rise to a soluble version of the receptor. A SNP within the exon 6 of *IL7R* has been identified in patients with multiple sclerosis that leads to skipping of exon 6 leading to increased levels of soluble IL-7Rα [122,123,124]. Splicing of the cytokine *Il7* gene has also been observed and various spliced forms of the IL-7 have been identified in granulomatous lesions of *Mycobacteria tuberculosis* patients and in transformed tissues and tumor cell lines [125]. Moreover, the study by Vudattu *et al.* [125], identified that skipping of exon 5 in the *Il7* gene led to increased STAT5 signaling via the IL-7R to promote T cell survival. More recent studies have identified an important role for pre-mRNA alternative splicing as a factor that contributes to the long-term survival of naïve CD4^+^ and CD8^+^ T cells in the peripheral immune system [26].

A novel allele of hnRNPLL was isolated through an ENU mutagenesis screen in mice to identify genes that regulate lymphocyte differentiation and homeostasis [126]. The novel allele was identified through genetic mapping in mice that displayed a significant decrease in CD4^+^ and CD8^+^ T cell numbers in the peripheral circulation [26]. The mutation was mapped to the *Hnrnpll* gene and was designated “thunder” for “T_h_ cells under the normal range” [26]. The mutation leads to a single amino acid substitution of a conserved valine residue for aspartate V136D within the RRM1 domain in the *Hnrnpll* gene. The valine residue is highly conserved in vertebrates and other similar domains in hnRNP proteins [26]. The V136D mutation did not affect binding affinity but rather greatly decreased the protein’s stability of the RRM1 domain leading to a loss of function [26]. Thunder mice have a 20 to 200 fold increase in CD45RA, RB and RC isoforms without increasing the overall surface expression of CD45 [26,127,128]. Furthermore, these mice cannot splice the *Ptprc* gene which gives rise to constitutive expression of the high molecular weight CD45RABC isoform in thymocytes and peripheral T cells within peripheral lymphoid tissue [26]. Initially at birth and in the first couple of weeks during postnatal development there is no significant difference in T cell numbers between wild type mice, but as the Thunder mice aged the CD44^l0^ (naïve) cell subset failed to accumulate normally in peripheral blood, spleen and lymph nodes. Furthermore there was a marked decline in naïve T cells by 4–6 weeks of age due to a T-cell intrinsic decrease in their persistence [26]. The demise of peripheral naïve T cells was compensated by an increase in CD44^hi^ memory phenotype T cells [26]. Interestingly, the lymphopenia observed in the hnRNPLL^thunder^ mice did not increase their chances of developing spontaneous autoimmune disease and when hnRNPLL^thunder^ mutation was bred onto a TCR_HEL_ × InsHEL double transgenic background the mice were not predisposed to developing autoimmune diabetes [26,127].

Initially it was thought that the lymphopenia in hnRNPLL^thunder^ mice was due to aberrant T cell signaling due to the lack of low molecular weight CD45 isoforms [26,127]. However, it was later shown that the hnRNPLL^thunder^ mutation disrupted peripheral T cell accumulation even in the absence of the CD45 protein [127]. It was concluded that hnRNPLL has a further role to play in regulating cell viability that is independent of its role in regulating CD45 alternative splicing. [127]. It would appear that the abundance of CD45 at the cell surface is more important for T cell differentiation in the thymus than the expression of different CD45 isoforms [127,129].

We further described that hnRNPLL seems to be more important to control the numbers of CD4^+^ and CD8^+^ T cells than other T cell populations. It was discovered that NKT cells accumulated normally in the thymus and periphery of hnRNPLL^thunder^ mice, despite the overlapping requirement for hnRNPLL in regulation of alternative splicing of the *Ptprc* pre-mRNA in NKT cells and TCRαβ^+^ cells [128]. Interestingly, the expression of developmentally regulated cell surface marker NK1.1 on NKT cells was significantly reduced in hnRNPLL^thunder^ mice [128], revealing again divergent roles for hnRNPLL in different lymphocyte subsets.

## 8. Alternative Splicing in B Cells and the Role of hnRNPLL in B Cell Development and Function

B cells with their ability to secrete a large amount of antibodies against various evolving pathogens are important players of the adaptive immune system. B cells develop in the bone marrow of most mammals in a highly regulated process that depends on cell intrinsic and extrinsic factors as well as the activation of transcription factors [130,131]. In order for B cells to generate a diverse repertoire of antigen receptors, Ig heavy and light chain rearrangements occur in precursor B cells in the bone marrow. Successful production of heavy and light chains allows precursor B cells to express IgM on the cell-surface. These cells, called immature B cells, are ready to leave the bone marrow and enter the periphery to complete their maturation. However, immature B cells need to be checked first for self-reactivity before they leave the bone marrow, if they express receptors with high affinity for self-antigens they must be either eliminated or silenced in order to maintain self-tolerance through several mechanisms which include receptor editing [132,133,134,135], clonal deletion [136] and anergy [137]. Immature B cells that pass the initial check-point begin expressing IgD on their surface and migrate to the spleen for their final stages of maturation into different subsets including follicular and marginal zone B cells [138]. The mature BCRs on B cells are in the form of membrane-bound Ig molecules and search for their cognate antigens during their circulation in the blood and lymph. When a BCR recognizes epitopes on a protein antigen the B cell becomes activated and differentiates into either an antibody producing plasma cell that secretes a soluble form of antigen specific Ig molecules to clear the pathogen, or differentiate into memory B cells to provide a faster and stronger immune response upon a secondary encounter with the same antigen.

There are five different antibody isotypes made by B cells namely IgM, IgD, IgG, IgE and IgA, which differ in their heavy chain isotypes produced from a single Ig heavy chain (Igh). These isotypes are produced at distinct stages of B cell lymphopoiesis and function as maturation markers, receptors (membrane-bound form), and effector molecules during the clearance of antigens (secreted-form). While IgG, IgE and IgA are produced by irreversible rearrangement of the gene after activation (called class switching), IgM and IgD are co-expressed through alternative splicing of a long primary mRNA transcript from the *Igh* locus [139,140], which is conserved in many species [141]. However, it remains unclear how alternative splicing of the *VDJ_H_* sequences to those exons encoding the constant regions of IgM and IgD is developmentally regulated during B cell maturation. The *trans*-acting factor hnRNPLL could be a candidate for controlling pre-mRNA processing during B cell development, but splenic B cells from hnRNPLL^thunder^ mice have normal IgM and IgD expression (unpublished data). This suggests that hnRNPLL is most likely dispensable for alternative splicing of *Igh*. Interestingly two independent groups recently identified zing-finger protein (ZFP) 318 to act as a regulator of IgD expression on mature B cells [142,143]. Mice deficient in ZFP318 exhibit a largely normal B cell phenotype, but all mature B cells fail to express IgD and display heightened IgM expression on the surface [142,143]. Consistent with its function in IgD production, mRNA levels of *Zfp318* in IgD^+^ B cells is higher compared to other IgD^−^ subsets [142,143,144]. These findings suggest the *Ighd* splicing is promoted during the maturation of B cells into IgD-expressing cells and this process is controlled by ZFP318 [142,143].

Upon activation B cells can differentiate into antibody-producing plasma cells and pre-mRNA splicing occurs to increase the synthesis of *Ighg2b* mRNA transcript encoding the secreted Ig [145], through alternative splicing of *Igh* genes at their 3′ ends [146,147,148]. However, the mechanism for posttranscriptional regulation of this process remains obscure. In order to identify pre-mRNA splicing regulator(s) of *Igh* in B cells, the Rao group compared the transcriptional profiles of bone marrow plasma cells *versus* naïve B cells. Both *Hnrnpll* mRNA and protein expression was significantly increased in plasma cells compared with naïve B cells [149]. Consistent with increased expression in plasma cells, the authors were able to identify the hnRNPLL binding sites within *Ighg2b* transcripts using PAR-CLIP. Given that the secreted *Ighg2b* mRNA is higher in plasma cells compared to naïve B cells with increased membrane-encoding *Ighg2b* [145], one can suggest that hnRNPLL might play a role in RNA processing to help generate the secretory-specific form of *Igh* mRNA *versus* membrane-bound form. However, the ratio of membrane *Ighg2b* to secreted *Ighg2b* was surprisingly reduced in a mouse plasmacytoma cell line that lacked hnRNPLL [149]. Thus, it is possible that the role of hnRNPLL in driving production of secreted *Ighg2b* in plasma cells somehow is masked and/or counteracted by other *trans*-acting factor(s). In keeping with this notion, the transcription elongation factor ELL2 has been shown to control accumulation of the secretory form of *Ighg2b* mRNA by plasma cells [149,150,151].

A report by Chang *et al.* [152], revealed that the role of hnRNPLL in B cell development and function goes beyond its impact on the regulation of *Ighg2b* transcripts. They used retroviral transduction of bone marrow cells that contained either shRNA knockdown of hnRNPLL or a control vector. The transduced bone marrow cells were transferred to irradiated recipients. B cell development in both groups of recipient mice was normal but the total number of mature B cells observed in the peripheral circulation of irradiated mice reconstituted with sh-hnRNPLL knock down bone marrow cells, was significantly reduced compared to control animals that received bone marrow containing the control sh-scramble [152]. These data suggest a possible role of hnRNPLL in the development of B cells in the bone marrow and/or periphery, which requires further examination. Moreover, hnRNPLL has also been shown to be involved in the differentiation of plasma cells *in vitro* and *in vivo* [152]. Since the differentiation of plasma cells from B cells is associated with exclusion of CD45 exons 4–6 [153,154], it is possible that hnRNPLL may control the switch to the CD45RO isoform during plasma cell differentiation. Indeed, in addition to its role in regulation of alternative splicing of exon 4, 5 and 6 in T cells, Chang *et al.* [152], showed that hnRNPLL selectively controls skipping of exon 4 during plasma cell differentiation. However, it remains unclear whether or not the inability to exclude exon 4 underlies the defect in plasma cell differentiation. Despite the evidence that hnRNPLL might mediate plasma cell differentiation it remains unclear if it can affect splicing of other master regulatory genes such as *Irf4* that are required for plasma cell differentiation [152,155,156], or whether hnRNPLL may have a more global effect on alternative splicing events to facilitate plasma cell differentiation.

Chang *et al.* [152], used the PAR-CLIP assay to reveal the hnRNPLL-binding sites on plasmacytoma RNAs that are related to the regulation of alternative processing of pre-mRNAs. HnRNPLL has been shown preferentially to bind to CA dinucleotide-containing RNA sequences in introns. Furthermore, 10% of the hnRNPLL-recognition sites were found to be within the 3′ UTR regions, which upon binding possibly promote mRNA stability [152]. Thus, these findings reinforce the critical role for hnRNPLL in alternative splicing of pre-mRNAs, and extend our understanding of the posttranscriptional regulation of mRNAs during the differentiation of immune cells upon activation.

## 9. Future Directions

The development of new molecular techniques have enabled scientists to learn more about the broader networks that regulate gene expression and alternative splicing within cells. The use of array technologies such as gene expression microarrays and exon arrays have uncovered the breadth of global changes to gene expression and splicing events that occur in response to cellular activation and differentiation. As previously highlighted by Martinez and Lynch [31] in order to gain a deeper understanding of splicing programs within specific cells there is a need to validate the splicing of predicted genes that are derived from the different assays by PCR or protein expression, which is something that has not been performed in all studies.

The use of cell based assay systems have demonstrated a strength to uncover key *trans*-acting factors that control alternative splicing in lymphocytes. However, the limitation of these studies is that it is difficult to examine how they would affect specific cellular responses to infection or immunization *in vivo*. The isolation of the hnRNPLL^thunder^ allele in a novel mouse strain provides a good example of how mutagenesis approaches can facilitate knowledge about the biological functions of regulatory proteins. Identification of the hnRNPLL^thunder^ allele helped to understand the physiological effects of altering a single splicing regulator and its impact on the immune system. The hnRNPLL protein plays a global role in regulating alternative splicing of CD45 in lymphoid and non-lymphoid cells, but its effects on cellular homeostasis appears restricted to naïve TCRαβ^+^ cells. This highlights the need for a broader understanding of the global signaling pathways that emanate from the surface via antigen and/or growth factor receptors and how these are coordinated to regulate the program of alternative splicing within a single cell. The biochemical signaling pathways that function downstream from the TCR, BCR, growth factor receptors and death receptors like FAS have been well described. Yet we still know little about how these key signals link the control of cell growth and survival to the activity of splicing factors. The use of conditional gene targeting and development of the CRISPR/Cas9 techniques for gene editing either *in vitro* or *in vivo* should also allow scientists to target specific hnRNP proteins within specific cell types to study their function. This could help to elucidate the roles of individual hnRNP proteins *in vivo* and uncover the broader global networks of interaction between proteins and genes that control alternative splicing. The recent studies highlighting the role of DNA binding nuclear factors and their effect on RNAPII stalling on exons highlights just how much more we have to learn about the molecular interactions that help control gene expression, alternative splicing and translation.

## 10. Conclusions

In conclusion, we have highlighted the important role for pre-mRNA alternative splicing in a number of key immune functions that can have an impact on the decision between immunity and tolerance. SR splicing factors and hnRNP proteins act as *trans*-acting factors to mediate exon inclusion or exon skipping of genes and their activity is tightly linked to cellular and environmental cues that impact on cell differentiation and maturation. The immune system relies on apoptosis to remove autoreactive cells and effector T cells at the resolution of infection. We have discussed how alternative splicing is used to control the activity of apoptotic effector proteins such as FAS, Bcl-X and Bim and how strength of antigen receptor signaling and other cell surface molecules such as CD45 and CTLA-4 can affect T and B cell responses. Clearly more needs to be learnt about the regulation in alternative splicing but as our understanding grows it may help scientists to develop therapeutic interventions where dysregulation in alternative splicing defects contribute to human disease that will not be restricted to just the immune system [157].
